# NeuroFlow: A General Purpose Spiking Neural Network Simulation Platform using Customizable Processors

**DOI:** 10.3389/fnins.2015.00516

**Published:** 2016-01-14

**Authors:** Kit Cheung, Simon R. Schultz, Wayne Luk

**Affiliations:** ^1^Custom Computing Research Group, Department of Computing, Imperial College LondonLondon, UK; ^2^Centre for Neurotechnology, Department of Bioengineering, Imperial College LondonLondon, UK

**Keywords:** FPGA, spiking neural network, neuromorphic, hardware accelerator, large-scale neural simulation, PyNN, STDP

## Abstract

NeuroFlow is a scalable spiking neural network simulation platform for off-the-shelf high performance computing systems using customizable hardware processors such as Field-Programmable Gate Arrays (FPGAs). Unlike multi-core processors and application-specific integrated circuits, the processor architecture of NeuroFlow can be redesigned and reconfigured to suit a particular simulation to deliver optimized performance, such as the degree of parallelism to employ. The compilation process supports using PyNN, a simulator-independent neural network description language, to configure the processor. NeuroFlow supports a number of commonly used current or conductance based neuronal models such as integrate-and-fire and Izhikevich models, and the spike-timing-dependent plasticity (STDP) rule for learning. A 6-FPGA system can simulate a network of up to ~600,000 neurons and can achieve a real-time performance of 400,000 neurons. Using one FPGA, NeuroFlow delivers a speedup of up to 33.6 times the speed of an 8-core processor, or 2.83 times the speed of GPU-based platforms. With high flexibility and throughput, NeuroFlow provides a viable environment for large-scale neural network simulation.

## Introduction

Reverse engineering the brain is one of the grand engineering challenges of this century. Various projects have been working on a number of aspects of this problem, including characterizing neuronal types and their genetic transcription (Hawrylycz et al., 2012), developing genetic tools for targeting individual cell types for probing or perturbation (Madisen et al., 2010; Kohara et al., 2013), recovering neural connectivity (“the connectome”; Livet et al., 2007; Oh et al., 2014), developing tools and computational infrastructure for large-scale neural simulations (Markram, 2006; Plana et al., 2007; Ananthanarayanan et al., 2009; Markram et al., 2011). With the development of neural simulators, neural modeling contributes to the advance in computer science research including the fields of artificial intelligence, computer vision, robotics, and data mining. The computational capability of brain-style computing is actively being investigated by several projects (Eliasmith et al., 2012; Furber et al., 2014) and new algorithms inspired by the principle of neural computation are being developed (Gütig and Sompolinsky, 2006; Sussillo and Abbott, 2009; Schmidhuber, 2014). Recently there is growing interest in building large-scale models using spiking neural networks (SNNs), which can achieve higher biological accuracy and more comprehensive functionality than smaller scale models (Izhikevich and Edelman, 2008; Eliasmith et al., 2012; Reimann et al., 2013). As a result, a number of computing platforms targeting SNNs such as SpiNNaker (Furber et al., 2013; Sharp et al., 2014), FACETS (Schemmel et al., 2010), Neurogrid (Silver et al., 2007), and TrueNorth (Merolla et al., 2014) have been developed to make large-scale network simulation faster, more energy efficient and more accessible. Neural simulation platforms generally make use of processors such as multi-core processors, Application-Specific Integrated Circuit (ASIC) chips, or Graphics Processing Units (GPUs), and offer various degrees of programmability, from programmable parameters to complete instruction level control. While ASICs have high performance and low power consumption, their architecture is fixed during fabrication and thus they lack the flexibility to adopt new designs or modifications according to user needs, such as precision of parameters, type of arithmetic representations, and the neuronal or synaptic models to be used. On the other end, GPUs provide some speedup over multi-core processors, and have good programmability and flexibility, but they tend to have large power consumption.

FPGAs have previously been used as an accelerator for neural simulation, both for conductance-based models (Graas et al., 2004; Blair et al., 2013; Smaragdos et al., 2014) and for point-neurons (Cassidy et al., 2007; Cheung et al., 2009; Rice et al., 2009; Thomas and Luk, 2009; Moore et al., 2012; Cong et al., 2013; Wang et al., 2013). High customizability, high scalability and fine-grained parallelism make FPGAs a good candidate for the development of a neural simulation platform. However, so far FPGA-based accelerators lack design automation tools, and support only a limited range of models, thus the resulting platforms often have relatively low flexibility. To provide neuroscientists a flexible neuromorphic hardware platform to simulate large-scale biological models using spiking neurons, we develop NeuroFlow, a reconfigurable FPGA-based spiking neural network simulator with a high-level API (Application Programming Interface) suitable for users with no prior knowledge of hardware or FPGA technology. It provides high speed-up in comparison to traditional multi-core or GPU simulators, while allowing developers and neuroscientists to implement models in a conventional software environment.

Compared to several other neural simulators which are implemented with customized hardware, NeuroFlow targets easy-to-maintain off-the-shelf systems and makes the neural platform more accessible and portable. It allows the developer to focus on optimizing the implementation of models and computations instead of building and maintaining the underlying hardware and system. Moreover, it is portable to other platforms. Thus, as newer and faster platforms become available, the performance of the platform can be increased without additional development effort by upgrading the hardware.

Unlike hardwired processors, using an FPGA provides the flexibility to change the models and computations in the neural simulation, allowing the platform to suit the needs of a large group of researchers. NeuroFlow stores neuronal and synaptic parameters in large off-chip DRAM (Dynamic Random Access Memory) modules to enable simulation of networks in the order of 100,000 neurons on a single FPGA. The size of network that can be simulated on a single processor is larger than a number of previous FPGA neural simulators such as those described by Graas et al. (2004) and Thomas and Luk (2009) which store all parameters using fast but small on-chip BRAM (Block Random Access Memory) modules. This system also has greater flexibility than approaches optimizing a specific class of neurons such as Cassidy et al. (2011) and Merolla et al. (2014).

Our platform has a number of novel aspects in comparison to existing simulators:

- We use PyNN to develop a high-level API which provides users with a model description and simulation environment. It is the first FPGA-based simulator to support the use of PyNN to configure the hardware processor and to automate the hardware mapping of neural models. This means that users do not need special hardware knowledge to use the accelerator, allowing them to benefit from flexibility and performance of specialized hardware with only software effort without the need to understand the details of the low-level implementation.
- NeuroFlow can automatically determine hardware design parameters based on the neural model used in the simulation to optimize the hardware resources, such as the degree of parallelism and the number of FPGAs to be used. It increases the performance and reduces the effort and time to use the hardware processor for researchers with little or no prior knowledge in hardware systems.
- We implement spike-time dependent plasticity (STDP) using the host processor as a co-processor. It demonstrates the possibility of workload allocation based on the computation to achieve optimal performance for neural simulation.

## Materials and methods

### Overview of neuroflow

The aim of NeuroFlow is to simulate large-scale SNNs with flexible model options. We develop the simulation platform based on FPGA systems from Maxeler Technology. It has a number of advantages over existing hardware accelerators, including high portability, flexibility, and scalability owing to the design of the hardware system. We develop a compilation flow to translate a neural network description in PyNN to that used by NeuroFlow as an abstraction layer for the underlying hardware. The high level description also aids in automatically determining hardware parameters such as the degree of parallelism, the number of FPGAs to use, and the optimizations that require additional hardware resources. As in previous work, we use software synapses written in memory as look up tables instead of physical wiring between modules (Cheung et al., 2012; Moore et al., 2012; Wang et al., 2013), which allows large-scale networks to be simulated at high speed. We also employ a number of hardware design strategies to speed up and parallelize the computations in NeuroFlow as described in Cheung et al. (2012), such as time-multiplexing, pipelining, event-based spike processing, and low-level memory optimizations, in the design of NeuroFlow. Currently users are required to use systems from Maxeler Technologies, but the design principles of the system can be applied to other FPGA-based systems with sufficient memory storage.

### Hardware system

#### FPGAs for reconfigurable computing

**Figure 1:**
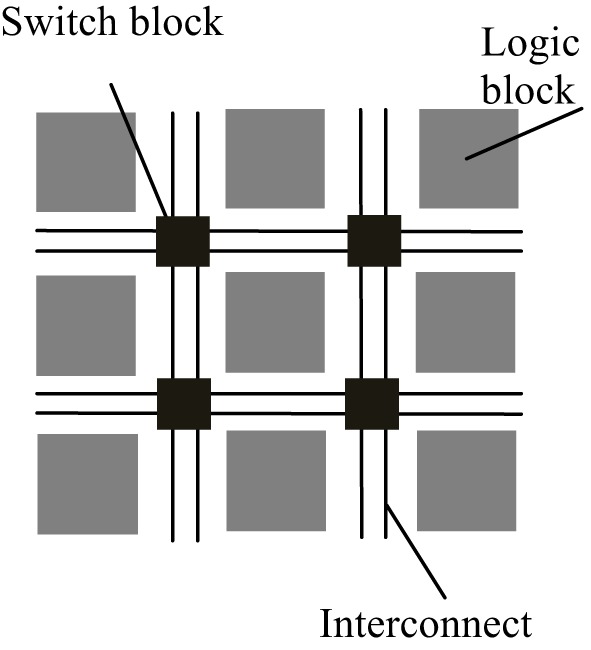
**Simplified architecture of a typical FPGA**. An FPGA is made up of a number of reconfigurable logic blocks, which implements arbitrary logic functions, and they are connected by switch blocks and interconnect wirings, which routes the input and output of logic blocks to desired destinations. The configuration of logic blocks and switch blocks is compiled by backend software using the functional descriptions written in hardware description language.

FPGAs are reconfigurable hardware processors consisting of arrays of configurable and fixed logic resources such as multipliers and on-chip memory modules. The logic resource blocks are connected via connection wires and switch boxes. The architecture of a typical FPGA is shown in Figure 1. FPGAs allow user-defined logic and computational operations to be implemented by configuring the logic resources and the circuit paths linking the input and output of the logic blocks, thus making the FPGA a customizable electronic chip with the ability to be reconfigured as desired. This flexible structure also makes it easier to parallelize the computations with lower performance overhead than general purpose processors. Although FPGAs typically operate at a much lower clock frequency than general purpose processors, customized computations implemented by the reconfigurable logic blocks mean that it may take only a few cycles to finish what may take a multi-core processor tens to hundreds of cycles to process.

FPGA offers a number of benefits for neural simulation compared to other processor types. The architecture of FPGA supports hardware reconfiguration which is capable of flexible functional descriptions. FPGAs have a large number of low-latency interconnects ideal for connecting processor nodes in a high performance computing system. They also enable greater control for memory access and fine-grained parallelization. Researchers have used FPGAs to develop neurocomputers for spiking and non-spiking artificial neural networks (Maguire et al., 2007). However, the difficulty of programming FPGAs can hinder their adoption for demanding applications such as neural simulation. Recently, high-level synthesis (HLS) tools capable of translating high-level languages into lower level descriptions have enabled faster development time, less effort for design modifications, and cross-system portability. For instance, in our system we use Java to describe the high level computation to be implemented on the chip.

#### High-performance FPGA-based computing platform

**Figure 2:**
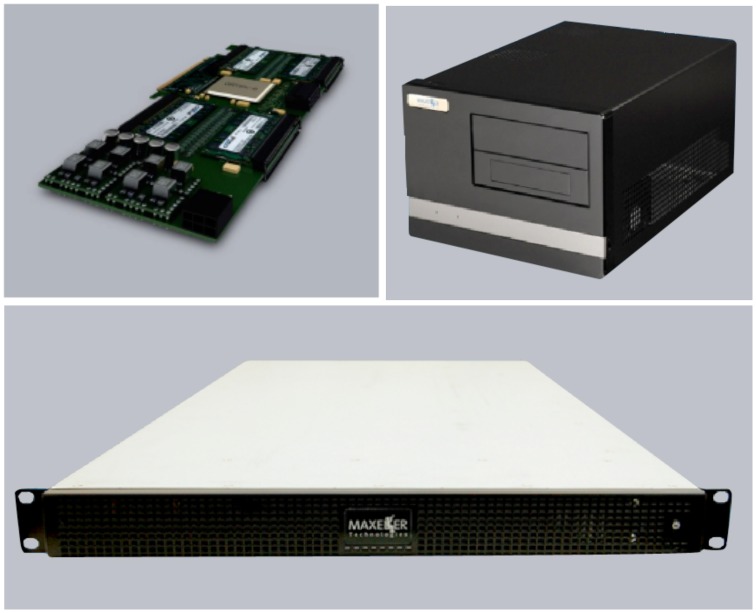
**Sample Maxeler systems the NeuroFlow neural simulator run on**. **Upper left**: A single system board containing one FPGA; **upper right**: workstation with a single FPGA card; bottom: a 1U server unit.

We choose systems provided by Maxeler Technologies as the targeted platform to develop NeuroFlow. The vendor offers a number of FPGA-based high-performance computing platforms. The FPGAs are configured as streaming processors, delivering high computing power with large external memory capacity. Different form factors are available including standalone desktops, multi-node server-scale systems, and on-demand cloud-based service. Figure 2 shows the system board and two of the available platforms. Similar to software platforms, the simulator can run on any of these form factors without modification if the underlying FPGA system board is of the same model. The various form factors have their own pros and cons and suit researchers with different needs: a standalone desktop provides affordable and exclusive access for researchers to simulate their own models; a server-scale system has a number of FPGAs in the same node and can be used for simulation of larger networks; cloud-based service on the other hand is more convenient and potentially cost less than the other options since it does not require ownership of physical systems, and is thus suitable for short-term deployment. In this work, we use a standalone desktop with 1 FPGA (Virtex 6, 40 nm process) and a rack server with 6 FPGAs (Altera Stratix V, 28 nm process) in our experiments. Since the compiler tools and the system architecture are customized for dataflow computing, NeuroFlow cannot be run directly on other standalone FPGA boards without modification.

### Simulator architecture and computation

#### Computation phases and core structure

**Figure 3:**
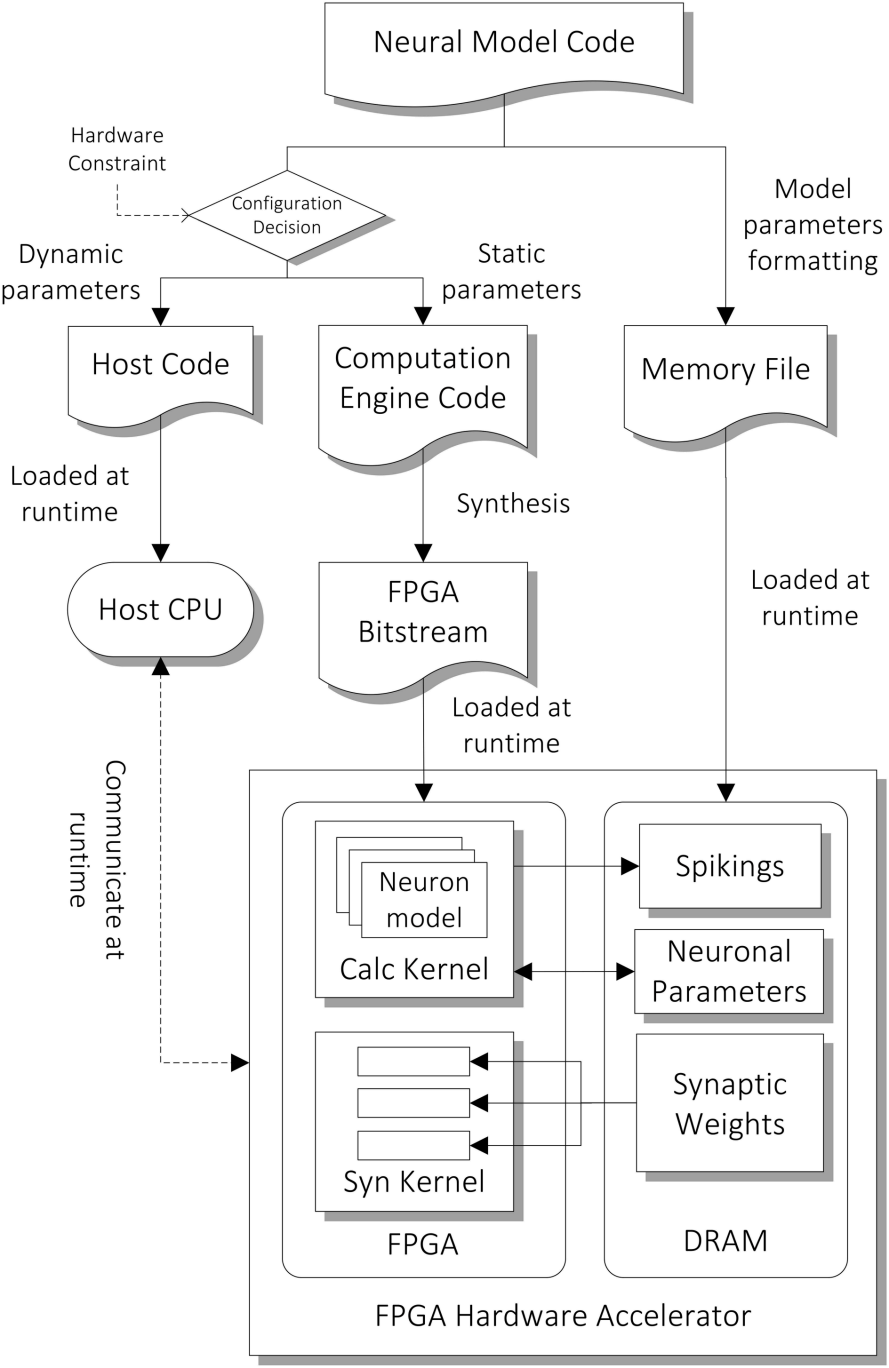
**Compilation pipeline and runtime file loading of NeuroFlow**. The pipeline receives neural model code written in Python which is then compiled into hardware configuration, host side configuration and memory data file. The resulting data files and configurations are loaded into the host processor (CPU) and FPGA of the system during runtime.

The architecture of the computation core is shown in the bottom part of Figure 3. The FPGA has two major hardware kernels which are synthesized from the high level descriptions, namely the Neuron State Kernel and the Synaptic Integration Kernel. Each of them is responsible for a phase in the two-phase computation. We use time-driven model for updating the state of neurons and each of the two-phase computation represents a time step of 1 ms. They communicate with the host processor node during the initial setup at the beginning of the simulation and during the read-out of the simulation results (spiking output) at the end of the simulation. During the initial setup, the architectural parameters and neuron model description are passed on to the hardware synthesis compiler, which initiates the compilation pipeline (Section Compilation Pipeline). The neuronal and synaptic parameters are then compiled and loaded onto the external DRAM on the FPGA card. During the simulation, the parameters to be retrieved for subsequent analysis designated by the user, such as spiking data and neuronal states of specified neurons, are stored on the external DRAM, and are transferred to the host after the simulation.

**Figure 4:**
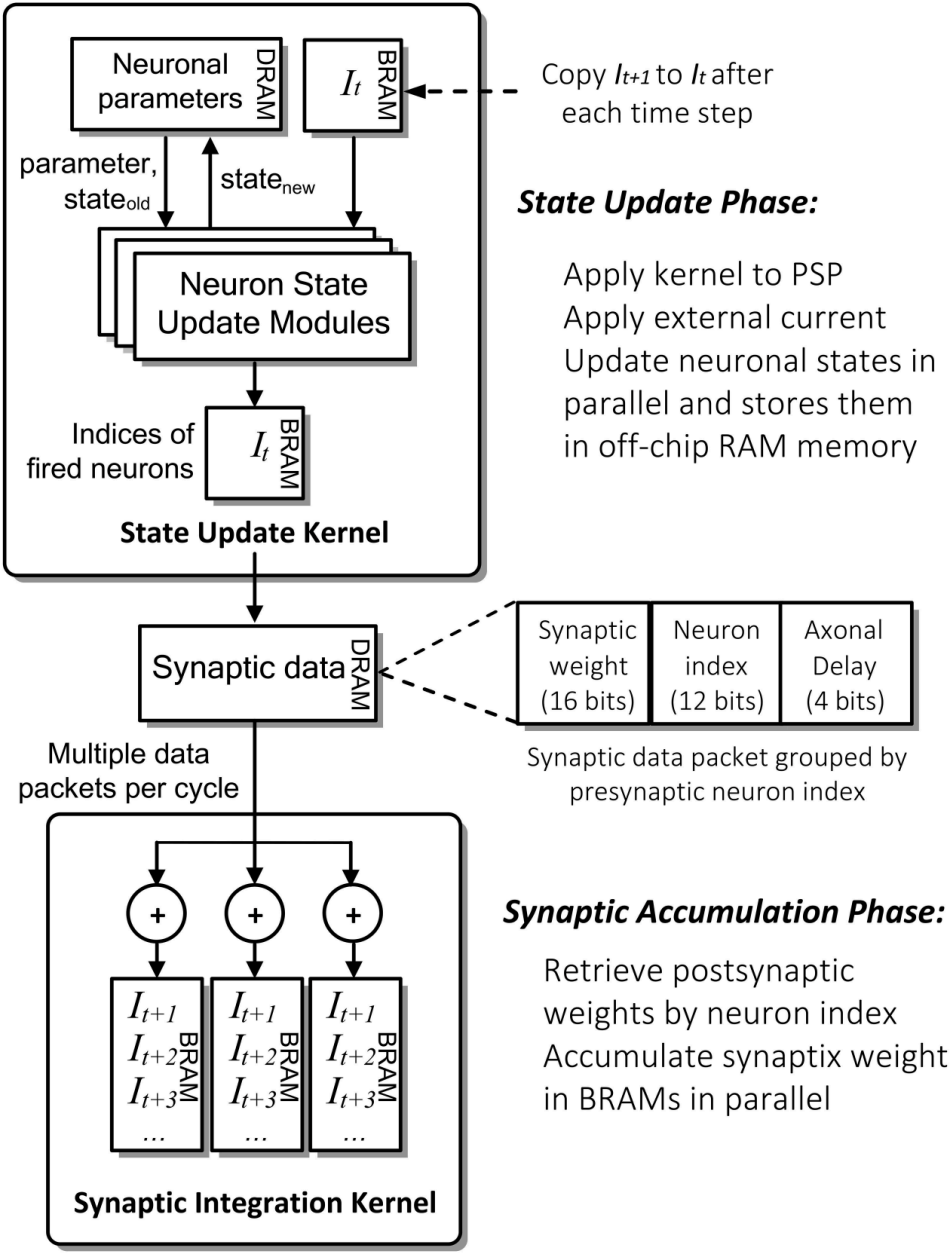
**Simplified computation flow and architecture of NeuroFlow**. The system consists of two major kernels corresponding to the two main computation phases: Neuron State Kernel, which corresponds to Neuron Update Phase and calculates the updated neuronal states using the synaptic and neuronal models at each time step; and Synaptic Integration Kernel, which propagates the neuronal spikes to own and other FPGAs in Synaptic Accumulation Phase. Neuronal and synaptic parameters are stored in high-capacity off-chip memory while the others are stored in high-speed on-chip memory to optimize the overall memory latency.

Figure 4 shows the detailed computation flow of NeuroFlow in the simulation phase. The main computation phases for SNNs are (i) neuronal state update and (ii) synaptic weight accumulation.

In the first phase of the process (i), FPGAs update the neuronal parameters and spiking record stored in large off-chip DRAM memory storage. They are retrieved and updated by the FPGA during the simulation. The neuron state update modules update the dynamics of neuronal states in parallel using ordinary differential equations-based neuronal models, which are computed in floating-point arithmetic. The number of parallel neuronal modules, typically ranging from 2 to 32, is determined by the number of neuronal parameters and memory bandwidth available. These customizable modules are implemented in a time-multiplexed, parallelized and pipelined manner to optimize the throughput with a range of model options to choose from.

In the second phase (ii), the FPGAs retrieve and store the incoming synaptic data into the fast on-chip BRAM memory storage. The Synaptic Integration Kernel retrieving synaptic data from off-chip memory and subsequently computes the synaptic input for the neurons. The computation is carried out by up to 48 parallel synapse modules, constrained by the available bandwidth and timing delay of hardware logic resources. Accessing synaptic data from memory and subsequent synaptic processing is triggered by spikes hence the overall processing time is roughly proportional to the activity level of the network. The synaptic data are preprocessed and packed in a specific format in fixed precision before being stored in the off-chip memory to speed up the simulation.

#### Nearest-neighbor STDP

**Figure 5:**
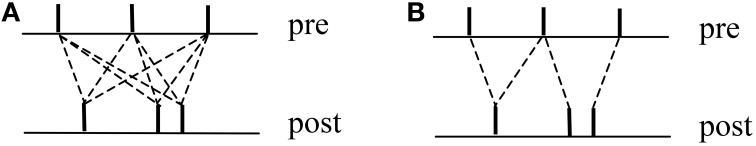
**(A)** All-to-all spike interactions between pre- and post-synaptic neurons for STDP learning. Since the number of interactions is proportional to the square of firing rate, this paradigm is computationally expensive. **(B)** Presynaptic-centric spike interactions between pre- and post-synaptic neurons for STDP learning adopted in NeuroFlow. This method is computationally efficient and can approximate the all-to-all paradigm with high biological plausibility.

Our simulator also supports nearest-neighbor STDP using both the FPGA and multi-core processors in a coordinated fashion. It demonstrates that conventional multi-core processors can be used along with the FPGA during a neural simulation in this system. Nearest-neighbor STDP is a common plasticity algorithm for learning in SNNs (Benuskova and Abraham, 2007; Babadi and Abbott, 2010; Humble et al., 2012). It is an efficient and good approximation of the STDP with all-to-all spike interaction as shown in Figure 5. It computes the change in synaptic weights using only the closest spike-pairs, instead of all spike-pairs, in order to reduce the computation complexity while remaining biologically accurate (Izhikevich and Desai, 2003). In accordance with Izhikevich and Desai (2003), we only take into account presynaptic-centric interactions such that only the first post-synaptic spike before or after a presynaptic spike can cause depression or potentiation, but nonetheless the system is capable of implementing other forms of interactions. We implement an additive model of STDP in NeuroFlow, and other types of more complicated models, such as a multiplicative synaptic model, can be incorporated, at the expense of requiring additional hardware resources. The hardware resource required would be proportional to the degree of parallelism of the synapse modules.

To compute STDP, the FPGA sends the spiking data to the multi-core processor which updates a table of relative spike timing stored on the host side with size proportional to the maximum axonal delay. The memory cache at the host side is more efficient for fetching and updating data in random memory locations, complementing the weakness of FPGAs in this respect. The relative timing information is used to access change in synaptic weights in a lookup table, which are accumulated and sent back to the FPGA. The communication for the update of synaptic weight table occurs at a fixed interval of time steps specified by the user (e.g., 100 ms and 1000 ms in the examples from the Section Results) to reduce the communication overhead.

To efficiently implement the STDP rule, we use a scheme similar to the deferred event model (Rast et al., 2009), which minimizes the number of memory accesses by batch processing the weight changes. The scheme stores spikes in previous time steps and calculates the corresponding weight change only after a certain time window, typically a few tens of milliseconds. The time window is set to ensure that no further spikes can cause changes to the synapses for a neuron spiking at the beginning of the window.

#### Automated customization using PyNN description

Since the FPGA platform is reconfigurable and has limited hardware resources, ideally one would simulate SNNs with a customized design for each specific neural model in order to optimize resource utilization. The high-level description of a network in PyNN facilitates automated customization to return a set of parameters for hardware design. We have identified a number of design parameters that can be determined by the simulation requirements and hardware constraints. Such customization provides further speedup at the expense of additional hardware resources. When the customization options are on, the compilation will attempt to compile the design with the highest hardware resource utilization, then proceeds to designs with lower resource requirements when there are insufficient hardware resources or the timing constraint is not met. The variables are divided into two types:

- Static parameters are compile-time parameters for the computation engine code, and recompilation of the hardware is needed whenever these parameters are changed. Since a typical recompilation of an FPGA hardware design requires 10–20 h, these parameters are not changed frequently for a given model and configuration. The static parameters are determined by analysis of the PyNN description and available hardware resources on the system. The parameters include:
  1. The number of parallel neuronal state update modules. In general the parallelism is calculated as the memory stream bit width divided by the precision (single or double precision) and the number of neuronal parameters per neuron.
  2. Additional weight caching mechanism for synaptic memory access. It makes use of extra on-chip BRAM buffers to store the data. Currently the additional resources required is estimated from previous builds. Together with the FPGA used and the network size to be simulated, the information can determine whether this mechanism can be accommodated in the design.
  3. Synaptic organization method to use based on density of neuronal connectivity. A densely-connected network can be packed in a more compact format which can lead to reduction in hardware resources.
- Dynamic parameters are runtime simulation parameters read by the host processor which do not need hardware recompilation. The parameters cannot be modified during a simulation but can be changed between different simulations in the same program. The parameters include the number of FPGAs to use, the allocation of simulated neurons and the number of neurons handled by each FPGA.

### Model specification

#### PyNN for neural model specification

PyNN is an open-source, Python-based neural simulation package for building neuronal network models developed by Davison et al. (2008). It offers an easy and standardized language for neural simulation and allows the results to be cross-checked between various simulation environments without the need to rewrite the code. Furthermore, users can also use visualization and analysis tools compatible with PyNN. Currently PyNN has API interfaces with a number of popular standard neural simulation software such as NEURON, NEST, and Brian (Davison et al., 2008), which can be installed along with the software packages. Hence the results from NeuroFlow can be crossed-checked with software simulators. A number of large-scale simulators have also implemented high-level bindings with PyNN to facilitate the use of the simulators such as SpiNNaker (Galluppi et al., 2010) and FACETS (Brüderle et al., 2011).

Taking advantage of its popularity, we adopt PyNN to provide a standardized interface for building neuronal models. Code 1 shows an example that can be used for declaring neuronal populations, creating neuron connections and setting hardware parameters in NeuroFlow. The user can define populations of neurons which share the same neuronal parameter(s) and connect them using various projection methods to add synapses. The user can also set hardware configuration parameters such as clock speed and degree of parallelism by using the setup command. If not explicitly specified, default values are used.

**Code 1:**
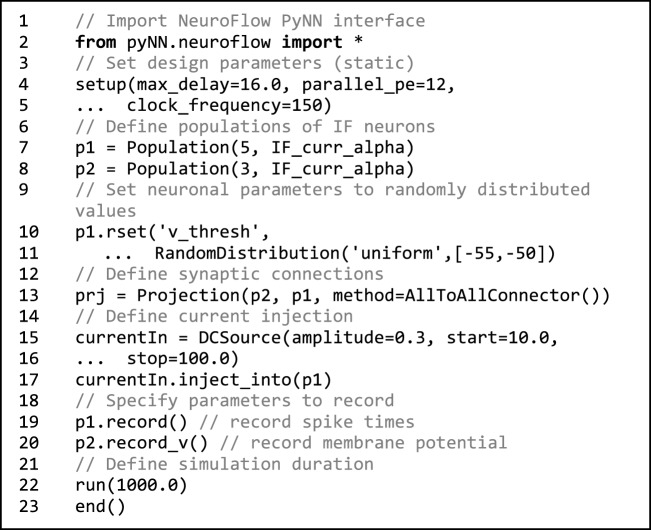
**Sample NeuroFlow PyNN interface code**. The usage is the same as the standard PyNN format. The code creates a number of integrate-and-fire neurons and connects them using an all-to-all connector method provided by PyNN. The user can supply external stimulation to the neurons and record the activity of neurons. The user can change the hardware configurations such as parallelism, maximum axonal delay, and FPGA clock frequency using the *setup* function.

#### Compilation pipeline

To automate the process of running neural models and determine hardware parameters, we develop a pipeline of compilation processes to translate a high-level specification to hardware configuration for the FPGA system.

The general compilation flow is depicted in Figure 3. In the first step of the pipeline, the PyNN interface, extracts, and converts the neuronal and synaptic parameters into a specific format as described in Cheung et al. (2012) to facilitate parallelization of neuronal state update and synaptic input accumulation, which is then written into memory data files. On the other hand, the compilation tool also determines a number of design parameters based on simulation conditions and hardware constraints to allow for customization and optimization of hardware resource (Section Automated Customization using PyNN Description).

In the next step, we make use of tools provided from Maxeler to compile code written in Java describing the computation and architecture into hardware descriptions readable by the FPGA. The compiler reads in the static parameters and chooses which models to implement in the synthesis phase according to user specifications. Instead of soft processors which are used by a number of other FPGA approaches (Maguire et al., 2007), the differential equations are compiled into dataflow path representations for building pipelined computations on the circuitry level. Although this approach is area-efficient, a fresh hardware compilation is required when a different neuronal model is used. The compilation tool then calls vendor-specific software which carries out conversion of lower-level hardware language descriptions in VHDL format, hardware resource mapping and place and route (PAR) which determines the actual hardware implementation. At the end of this process, an FPGA bitstream file is generated which describes the hardware configuration for the simulator. The host processor then reads in the host code generated from the compilation process and initiates the communication with the FPGAs and starts the simulation process.

#### Levels of description

To build a generic neural simulation system that enables the neural modeling community to use this accelerator as a simulation back-end, the actual hardware implementation should be abstracted from the user. We use PyNN and Maxeler tools to implement a number of abstraction levels for running neural simulations.

**Figure 6:**
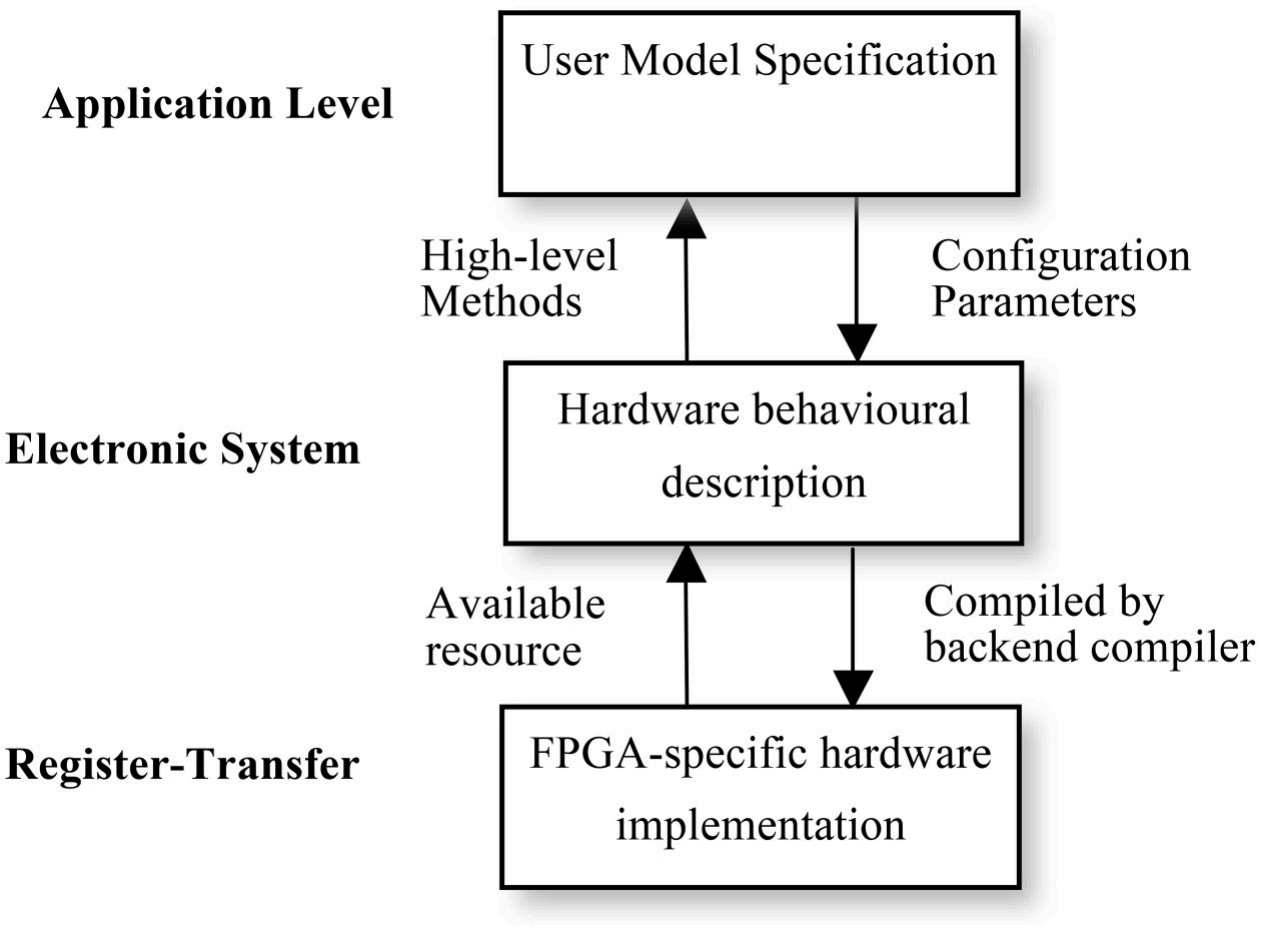
**Levels of description for neural simulation in NeuroFlow**. The description of the system of NeuroFlow consists of a number of levels: Application level, electronic system level and register-transfer level. Users of the system only codes in the application level and use functions provided from the levels below, without the need to know the hardware details. Changes to the actual implementation of neural models are made at electronic system level which is hardware independent. The register-transfer level is the implementation at the hardware level and is handled by software provided by the vendor, thus reducing the maintenance effort for the system.

Using multiple levels of abstraction, NeuroFlow allows users to write in a domain-specific language they are familiar with to obtain hardware speedup without extensive knowledge of hardware. As shown in Figure 6, each description level of NeuroFlow describes a separate aspect for simulating neural network on FPGAs. Each level can be modified independent of other levels and enable easier maintenance and development for the platform.

The top application level describes the neuronal types, network connectivity, and experimental setup. The description will be portable to various back-ends for checking and verification. It corresponds to the user code in Python in NeuroFlow, where user specifies the neuronal model and the customization parameters, and the translation work for extracting the description in Python to the subsequent stages in the pipeline. Next, the electronic system level describes the behavior of computation flow (differential equations, synaptic integration and STDP) and the system configuration, such as off-chip memory and inter-processor communication. When a different system is used, only the system configuration needs to be changed, rather than the entire computation engine code, thus facilitating portability across various systems. The hardware is also automatically customized based on the high-level description, meaning that performance gain is obtained without compromising the usability of the system. At the lowest FPGA-specific hardware description level, the program is translated to a hardware description language such as VHDL, which is then compiled to the actual FPGA bit stream using back-end vendor specific synthesis tools.

#### Supported simulator functions

NeuroFlow offers a number of common models and functionalities used in neural simulation which is summarized in Table 1. Currently a number of neuronal models are supported by NeuroFlow, and the process of adding new models to the simulator is relatively simple, by adding the needed neuronal options in the PyNN backend and writing the corresponding differential equations in the Java functional description. The models currently supported include the leaky integrate-and-fire model (Gerstner and Kistler, 2002), the adaptive exponential integrate-and-fire (Brette and Gerstner, 2005), the Izhikevich model (Izhikevich, 2003), and the Hodgkin-Huxley model (Hodgkin and Huxley, 1952). Furthermore, the simulator can use various ordinary differential equation (ODE) solvers; currently, Euler and Runge-Kutta methods are implemented in NeuroFlow to demonstrate this capability. The neurons can be divided into neuronal populations and neuronal parameters that are common in a population can be declared through the PyNN interface, and thus reduce memory usage by removing duplicated parameters.

**Table 1:** **Summary of functionalities supported by NeuroFlow**.

Module type^*^	**Implemented models**
Neuron models	• Leaky integrate-and-fire • Adaptive exponential integrate-and-fire • Izhikevich model • Hodgkin-Huxley model
ODE integration methods	• Euler method • Runge-Kutta method (4th order)
Synaptic input current filters	Exponential decay function • Alpha function • Custom filter
Plasticity	• Pair-based nearest-neighbor STDP
Simulator functions	• Neuronal population declaration • Neuron monitor (spikes and membrane potential) • Synaptic coupling strength retrieval (per presynaptic neuron) • External current injection • Random number generator (Gaussian/uniform)

* *Planned support for non-linear synapse dynamics and ball-and-stick neuron model*.

The current scheme assumes synaptic weights are linear and additive for both excitatory and inhibitory synapses, which are accumulated in the on-chip memory, and can be changed if required. The user can define various synaptic current input functions, such as exponential decay function, alpha-function, or delta-function.

The user can specify arbitrary external current injection to the neurons, and the parameters such as amplitude, time, and target neuron index are stored in on-chip memory. A number of random number generators are available to generate noise input to the neurons, including uniform and normally distributed random number generators. The user can also monitor the spiking and membrane potential of specified neurons, which are stored on FPGA during the simulation, and are sent back to the host processor after the simulation is completed.

The current implementation of the system maps the neurons into hardware by sequentially assigning the neurons to the FPGAs according to their indices. While this approach is simple and efficient, the communications between the FPGAs may not be optimized and could lead to longer simulation time when using more than one FPGA. In theory the connectivity between the FPGAs and the mapping can be done in a similar manner as Furber et al. (2013) which introduced a virtual mapping layer for efficient spike transmission.

## Results

To evaluate the performance of the platform, we simulate a number of models to test the accuracy, speed, and power consumption of our platform. The models vary in size and complexity to test the full range of capability offered by NeuroFlow.

### Two neurons with paired stimulation

**Figure 7:**
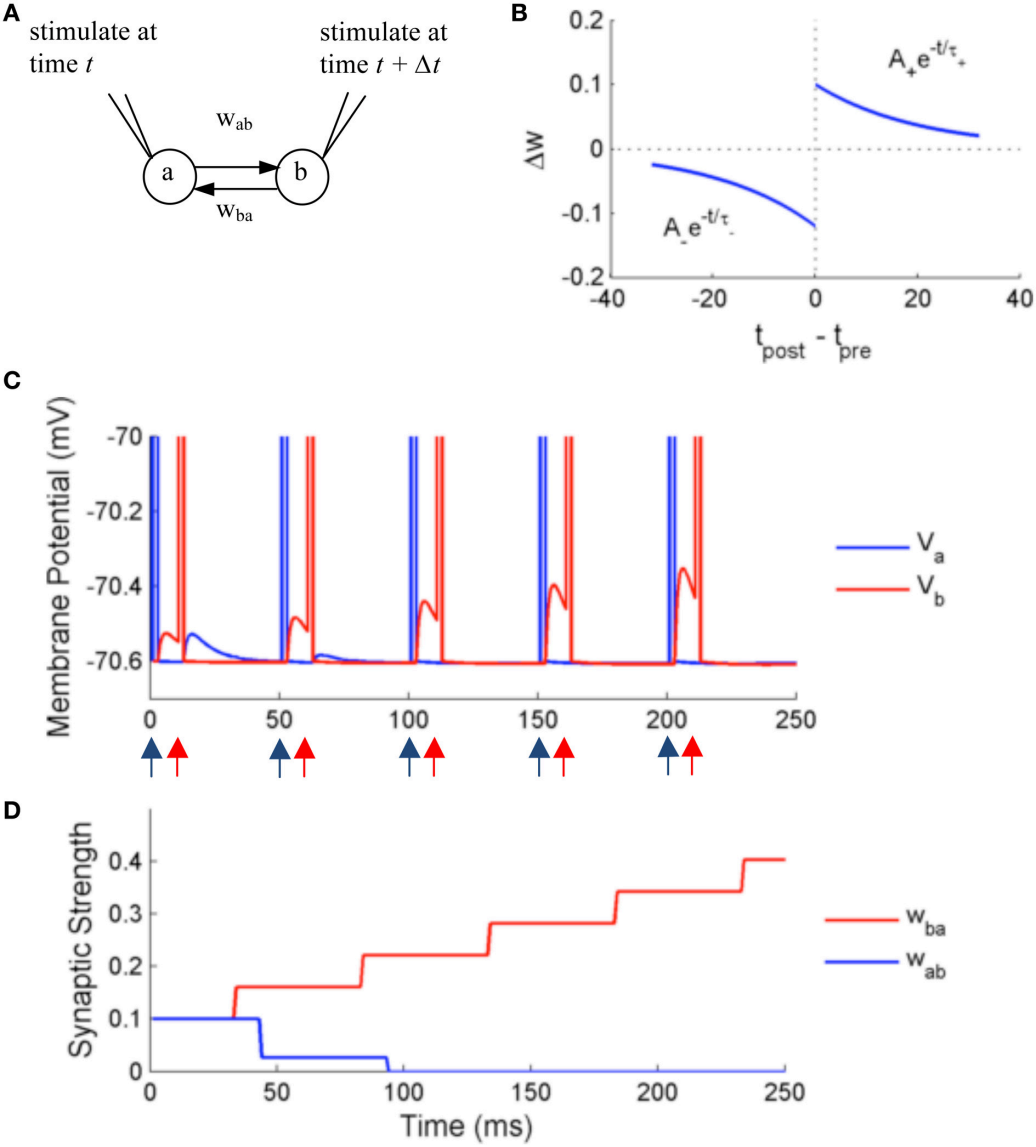
**Simulation using NeuroFlow with STDP enabled under the paired stimulation protocol**. **(A)** The simulation consists of two aEIF neurons which are mutually connected. Each of them receives an alternate stimulation of 10 mA for 250 ms. **(B)** The STDP profile used for the simulation, with A_+_ = 0.1, A_−_ = 0.12 and τ_+_ = τ_−_ = 20 ms **(C)** Traces of membrane voltage of the two neurons with spike time difference of 10 ms. The neurons spike upon receiving the external stimulation, where blue (red) arrows correspond to the time when neuron a (neuron b) receives external stimulation in the diagram. **(D)** Evolution of the strength of synaptic coupling between the two neurons across time in the trial described in **(C)**.

In the first set of experiments, we test the precision and accuracy of the computations by simulating two adaptive exponential integrate and fire neurons (aEIF) with external stimulation and STDP (Figure 7A). The membrane potential dynamics is described by the following equations:

(1) $$\text{C}\frac{\text{dV}}{\text{dt}}=-{\text{g}}_{\text{L}}(\text{V}-{\text{E}}_{\text{L}})+{\text{g}}_{\text{L}}\Delta_{\text{T}}\text{exp}\left(\frac{\text{V}-{\text{V}}_{\text{T}}}{\Delta_{\text{T}}}\right)-\text{w}+\text{I}$$

(2) $$\tau_{\text{w}}\frac{\text{dw}}{\text{dt}}=\text{a}(\text{V}-{\text{E}}_{\text{L}})-\text{w}$$

The model is a system with two differential equations, which solves for membrane potential V and adaptation variable w. The model receives input current I, and has parameters membrane capacitance C, leak conductance g_L_, reversal potential E_L_, spiking threshold V_T_, slope factor Δ_T_, adaptation coupling parameter a, and adaptation time constant τ_w_. Brette and Gerstner (2005) have shown that the model is able to replicate the precise temporal dynamics of biological neurons.

We test the effect of STDP on the synaptic strength by simulating two neurons firing alternately for duration of 250 ms, using an external input current of 10 mA with spiking time difference of 10 ms. A common STDP profile with temporal causality is applied (Figure 7B). The weight is modified according to the following rules:

(3) $$\Delta {\text{w}}_{+}={\text{A}}_{+}\text{exp}\left(-\frac{\Delta \text{t}}{\tau_{+}}\right)\text{ for }\Delta \text{t}>0$$

(4) $$\Delta {\text{w}}_{-}={\text{A}}_{-}\text{exp}\left(-\frac{\Delta \text{t}}{\tau_{-}}\right)\text{ for }\Delta \text{t}<0$$

with parameters A_+_ = 0.1, A_−_ = 0.12, and τ_+_ = τ_−_ = 20 ms, where only the closest spikes for each firing from presynaptic neurons are considered.

Figures 7C,D show the results of the simulation. The neurons are stimulated externally to force the neurons to fire at the times denoted by the red and blue arrows. The two neurons produce excitatory post-synaptic potentials (EPSPs) in the other neuron when they spike, with an initial synaptic strength of 0.1. Due to the effect of STDP learning, neuron B receives stronger synaptic input from neuron A, and conversely neuron A receives weaker synaptic input from neuron B. The synaptic strength is bounded to user-defined values, in this case 0 < w < 0.25, thus w_ba_ is settled at 0 after learning. The update of synaptic strength is delayed for the routine to take into account the timing of future spikes, in order to update synapses in batch.

### Large-scale spiking network

**Figure 8:**
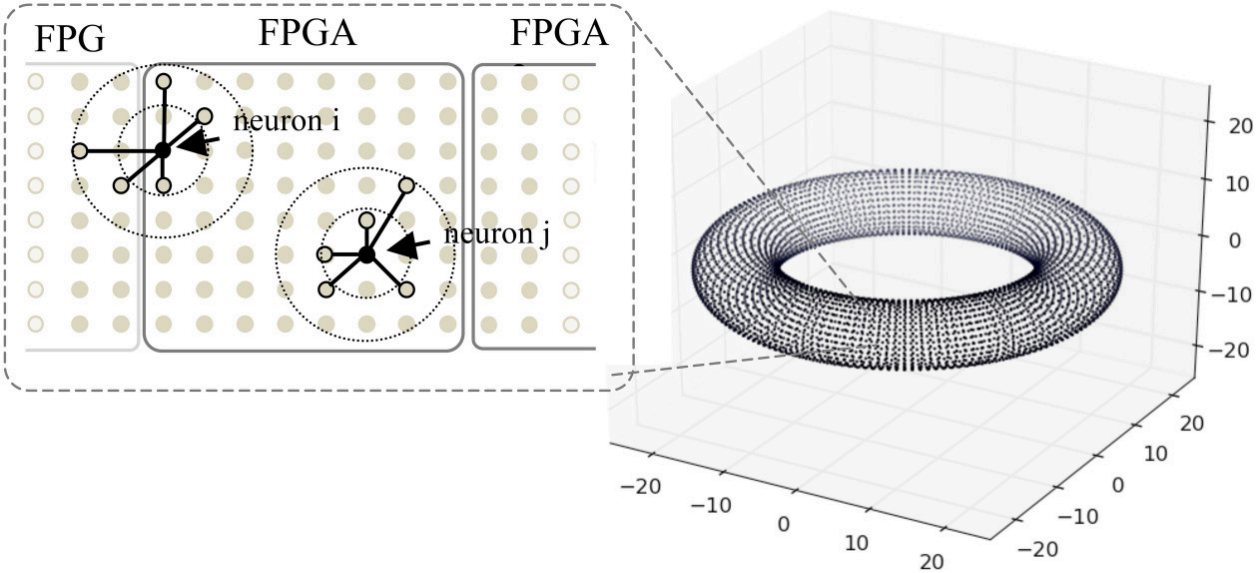
**A toroidal neuronal network**. Each dot represents the position of a neuron. Synaptic connection probability depends on the distance between the neurons which has a Gaussian distribution. Dotted circles denote connection probability within 1 and 2 S.D. One FPGA handles the computation of 98,304 neurons.

To test the performance of NeuroFlow for simulation of large networks, we simulate large-scale networks of size up to 589,824 neurons with excitatory and inhibitory neurons as shown in Figure 8. The network is similar to the one used by Fidjeland and Shanahan (2010), with neuron parameters taken from Izhikevich (2003), for evaluation of their neural simulator. The following table shows the hardware and simulation details. The setup time and readout time stated increases with the size of the network, while the readout time increases with the simulation time.

**Table:** 

FPGA Model	Stratix V (5SGSD8)
DRAM Memory	DDR3 48 GB
FPGA Clock Frequency	145 MHz
DRAM Clock Frequency	533 MHz
Power (per FPGA card)	30–40 W
Hardware Compilation Time	17–20 h
Setup time (for network in Figure 8)	< 2 min
Readout time (for network in Figure 8)	< 1 min

The network has a toroidal structure and the connection probability is spatially constrained. Each neuron connects to varying number of post-synaptic neurons n_syn_ with either static or plastic synapses, to test the effect of synapse numbers on the platform performance (in this case without STDP, i.e., for static synapses), where n_syn_ ranges from 1000 to 10,000 in the simulations. The synaptic strength values are set to random values between 0 and 0.5, and are adjusted by a scaling factor n_syn_ in order to produce a similar level of network activity across scenarios of different n_syn_. The connection probability follows a Gaussian probability of the synaptic distance, with standard deviation (S.D.) of varying σ for connections from excitatory neurons, and S.D. of 16 for inhibitory neurons. We test σ ranging from 32 to 512 to evaluate the effect of connection sparsity on the performance of the system. Conductance delays of the synapses are proportional to the distance between neurons, with a maximum of 16 ms delay for excitatory synapses and 1 ms delay for inhibitory neurons to ensure fast inhibition from the inhibitory neurons and rhythmic activity of the network.

When mapping the network onto FPGAs, each FPGA handles the computation of 98,304 neurons with close spatial proximity, and a maximum of six FPGAs are used for the simulations. Due to the locality of synaptic connections, the neurons connect to targets on the same or neighboring FPGAs, thus the hardware mapping of neurons facilitates the retrieval of synaptic data. Neurons that are closer to the border, such as neuron *i* in Figure 8, require slightly more time to gather synaptic data than neuron *j*.

**Figure 9:**
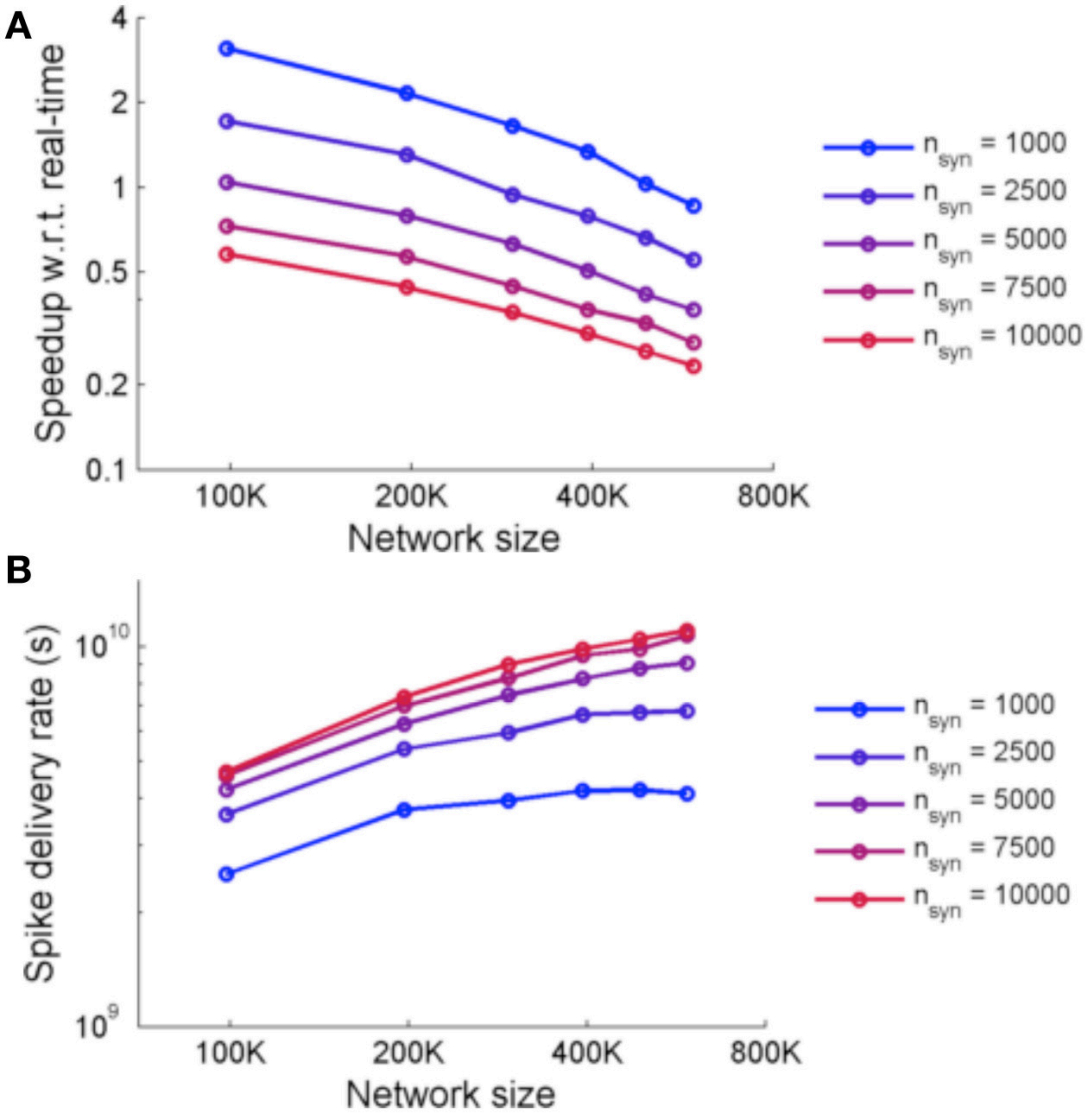
**(A)** Speedup of NeuroFlow with respect to real-time and **(B)** performance of NeuroFlow in terms of spike delivery rate. The speedup of NeuroFlow is inversely proportional to the size of the network, and networks with smaller number of synapses per neuron run a number of times faster. Due to overhead of distributing the computations to different processors, the speedup of network is not linear to the size of the network.

Figure 9 shows the performance of the system for simulations using two metrics: speedup with respect to real-time and spike delivery rate. Speedup with respect to real time is affected by the number of FPGAs required and the number of synapses. The spike delivery rate, suggested by Fidjeland and Shanahan (2010) as a measure of performance which measures the throughput of the system irrespective of the firing rate, is obtained from calculating the number of spikes delivered per second by the system.

**Figure 10:**
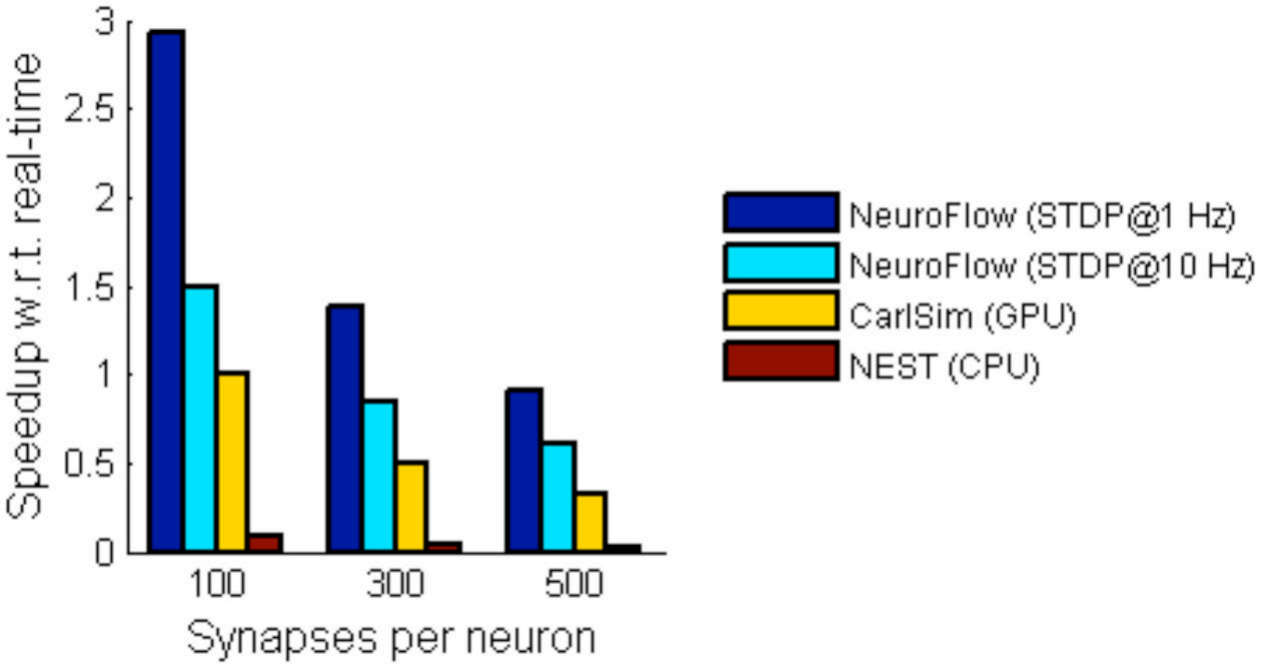
**Comparison of performance of various simulators**. The performance of NeuroFlow is compared against that of a CPU-based software simulator NEST and a GPU-based simulator CarlSim. NeuroFlow is faster than GPU and CPU platforms by 2.83 and 33.6 times respectively, when simulating networks of 55,000 neurons using 100, 300 and 500 synapses, with STDP enabled using an update frequency of 1 Hz.

For simulations involving STDP, we simulate a network with 55,000 neurons with STDP applied to excitatory synapses using the STDP profile in Section Computation Phases and Core Structure. The inhibitory synapses are set as non-plastic as described in previous literature (Phoka et al., 2012). We test two cases with different synaptic strength update frequency, 1 and 10 Hz, and measure the performance. The frequency determines the interval between the batch update of synaptic weights, and hence a lower update frequency incurs less overhead. The weight changes are batched and then updated to the FPGA DRAM in every fixed interval (1 s and 100 ms, respectively). Figure 10 shows the performance of NeuroFlow in comparison with multi-core and GPU simulators. Since the total number of synaptic updates is proportional to the update frequency of STDP and the synapse numbers, the computation time is heavily influenced by these two parameters. Updating the synaptic weights in FPGA also require a large bandwidth for the data transfer between the host processor and the FPGA, which limits the update frequency of the weights. We compare the performance of NeuroFlow to those of software simulator NEST (Gewaltig and Diesmann, 2007) on an 8-core i7 920 processor and GPU-based simulator (Richert et al., 2011) on a Tesla C1060 GPU. Using an update frequency of 1 Hz, NeuroFlow is on average 2.83 times faster than a GPU and 33.6 times faster than a conventional processor.

### Polychronous spiking neural network

We simulate a network that exhibits polychronization as demonstrated in Izhikevich (2006) as a form of functional verification. Making use of the interplay between conductance delay and STDP, the model displays a large number of self-organized neuronal groups which exhibit reproducible and precise firing sequences depending on a specific activation pattern (**Figure 12A**). The number of patterns can be larger than the number of neurons in the network, showing the potential computational capacity spiking neurons have over the traditional rate-based neuron models. The network also exhibits a range of rhythmic activity and a balance of excitation and inhibition similar to biological networks. Due to the long simulation required to demonstrate the effect of STDP on the firing patterns, it is a good example to demonstrate the need for neural simulation platforms such as NeuroFlow and other FPGA-based implementations such as that by Wang et al. (2013) for such experiments.

In the experiment, we simulate a network of 1000 Izhikevich neurons each with 100 plastic synapses. The simulation runs for 24 h in model time with 1 ms temporal resolution. At each millisecond, one neuron is randomly chosen in the network and receives a random input of amplitude 20 mV. The connection probability is 0.1 between neurons and STDP is enabled for excitatory synapses. Each synapse has a random 1–20 ms conductance delay. The simulation takes 1435 s to complete in NeuroFlow, which is 15 times faster than the 6 h reported in the original study using a PC with a 1 GHz processor. Various combinations of presynaptic anchor neurons in the absence of the random input are tested after the simulation to find out possible neuronal groups.

**Figure 11:**
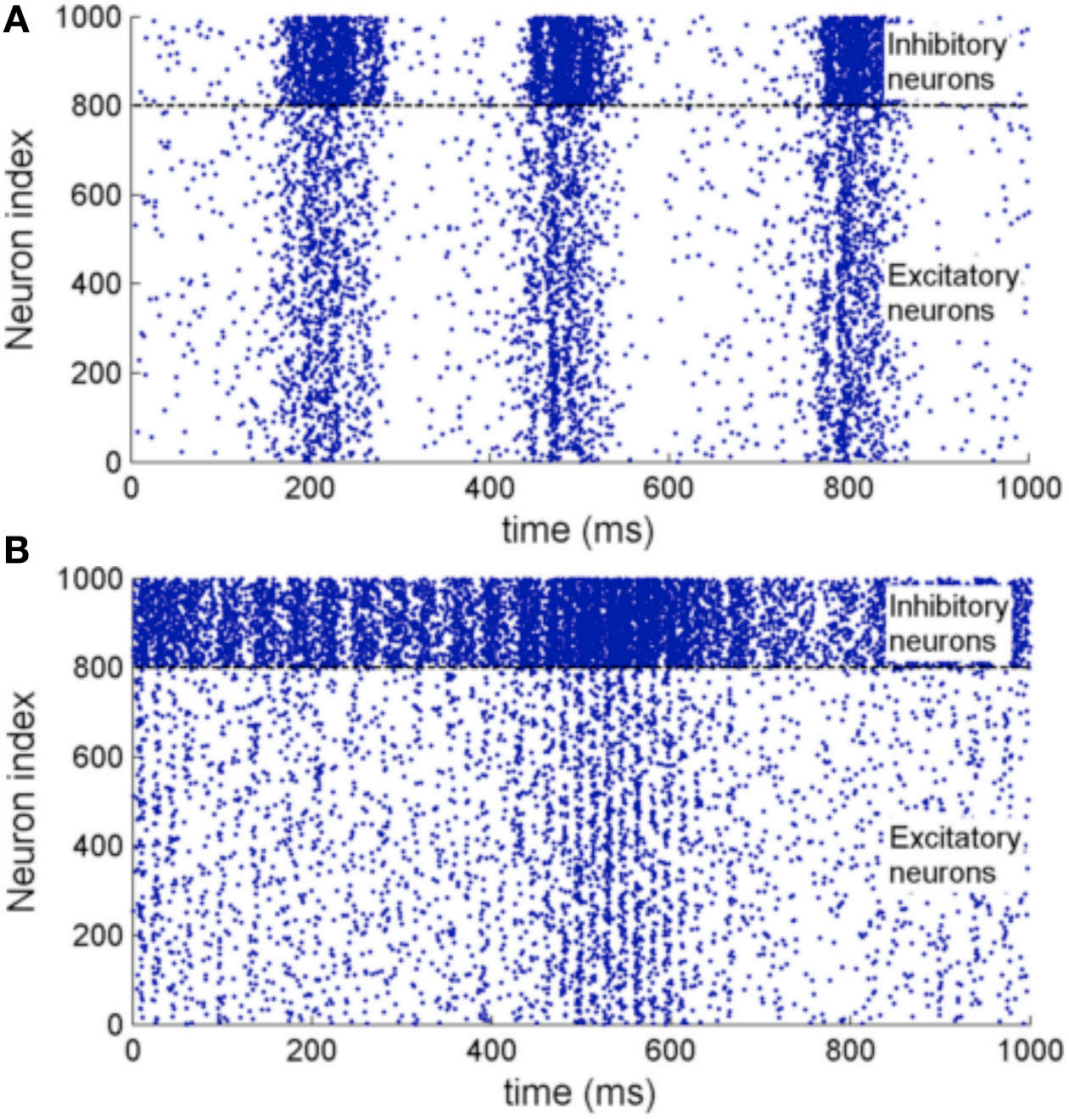
**(A)** Raster plot of the network activity with 1000 neurons at the beginning of the simulation. **(B)** Raster plot of the same set of neurons after a 3600 s simulation with STDP enabled. It shows oscillations of 2–4 Hz before the simulation and 30–100 Hz after the simulation which is of close resemblance of the original implementation.

**Figure 12:**
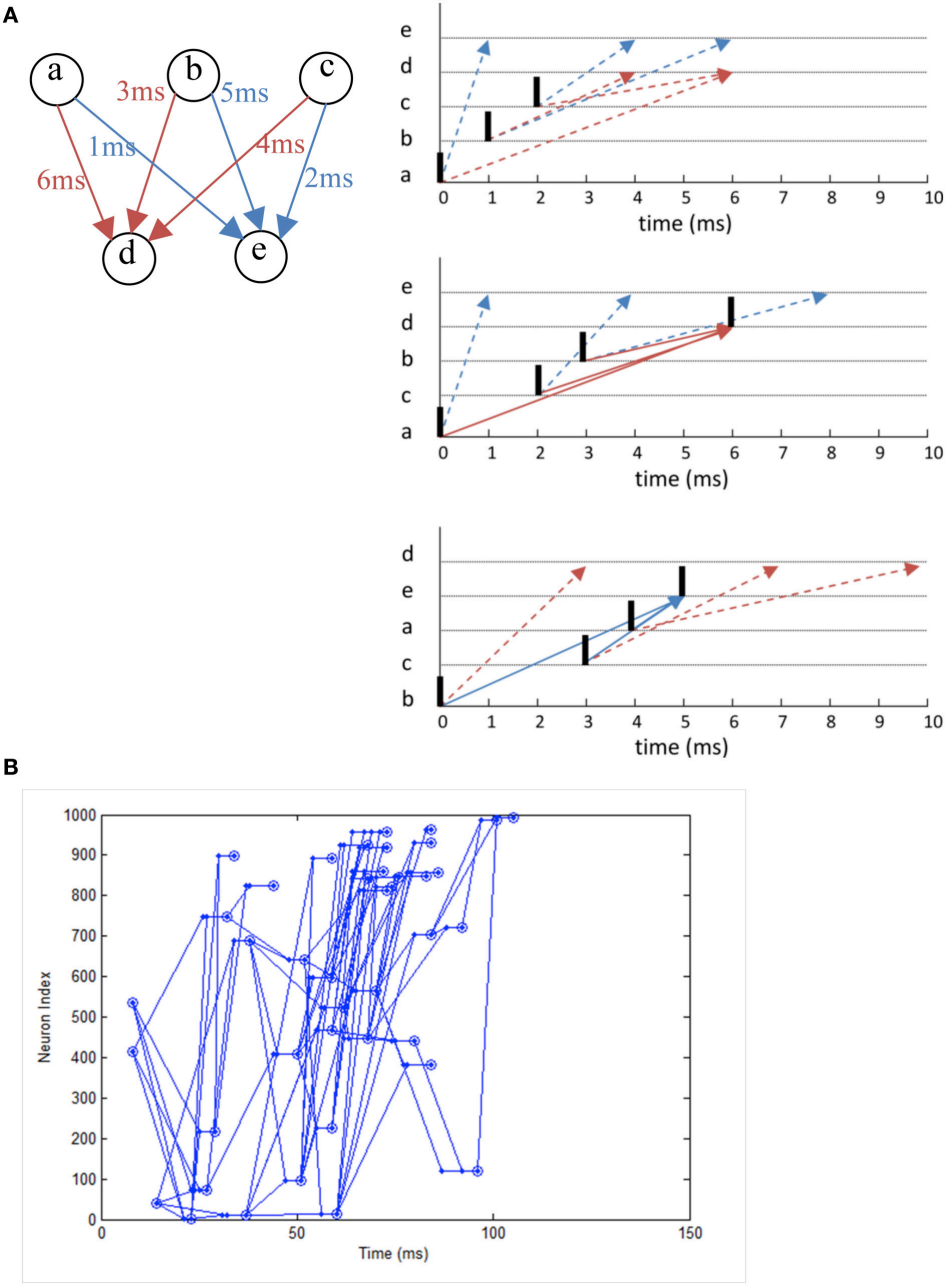
**(A)** The working principle of polychronization. Izhikevich (2006) proposed this mechanism, which shows that the various spike conductance delay can produce time-locked spiking patterns which are triggered by certain initial activation pattern. The pattern occurs spontaneously during long simulations of network with STDP. The dotted line represents the propagation of spikes in the network. **(B)** Polychronous network simulated produces a number of time-locked patterns upon activation of certain neurons.

Figure 11 shows the raster plot of the simulation to demonstrate the effect of STDP on the neuron dynamics. A 2 to 4 Hz rhythm is seen in the network when the simulation first started, which disappears during the simulation and is replaced by a gamma rhythm of 30–100 Hz after a simulation time of 1 h in model time. Figure 12B shows a sample neuronal group after the simulation. The neurons spontaneously self-organize into groups and generate time-locked patterns. Post-simulation analysis finds 520 neuronal groups which is consistent throughout the whole simulation after the first modeling hour. The simulated data are in agreement with results from the original study, giving rise to a similar distribution of group sizes.

## Discussion

In light of the increasing interest of simulating large-scale neural networks, there is a need for platforms with such capability which are powerful but easy to use and flexible. Targeting customizable FPGAs, we develop NeuroFlow which offers a flexible, portable and scalable simulation environment for SNNs that is available in various form factors, ranging from multi-node supercomputers to standalone desktop systems. NeuroFlow seeks to fill a gap in terms of flexibility and scalability across the spectrum of customized SNN accelerators. At one end of the spectrum, ASIC-based systems have the highest speedup and lowest power consumption amongst all forms of neural computing systems (Silver et al., 2007; Schemmel et al., 2010; Merolla et al., 2014), but they lack flexibility and are not widely available due to high manufacturing cost. At the other end of the spectrum, accelerators based on multi-core processor and GPU systems support a wide variety of synaptic, neuronal and plasticity models (Fidjeland and Shanahan, 2010; Richert et al., 2011; Hoang et al., 2013), but the power consumption is high for a large GPU cluster. FPGA-based systems offer a middle ground which may be of great utility for large scale brain simulation programmes. Following on from the first initial FPGA implementations of spiking neural systems (Cheung et al., 2012; Moore et al., 2012; Wang et al., 2013, 2015), here we report in detail the first FPGA based, flexible, general-purpose implementation of a large-scale spiking neural network that includes “full” Izhikevich style neurons and STDP.

In the design of customized neurocomputers, flexibility is sometimes sacrificed to achieve higher speedup, but it makes the platform less useful for neuroscientists who usually carry out specific and rapid model modifications. ASIC designs have the lowest programmability and flexibility. The spiking neuron models are fixed during fabrication and synaptic connections are often non-plastic, low precision and have constraints in the number and patterns of connections. In comparison the SpiNNaker system, based on ARM processors, offers a high degree of flexibility, but due to the small number of neurons handled by each processor (at the order of 10^4^ neurons) and the requirement of long communication paths for large networks, the platform does not handle well cases with a large number of events, such as dense connectivity, STDP, or a highly active network (Furber et al., 2014). These situations are commonplace in neural simulations but are often not considered when building neuromorphic systems. Furthermore, customized hardware platforms have to be redesigned and fabricated when there is a need to replace or incorporate additional models, such as plasticity, neuronal compartments, ion channels, gap junctions, or higher precisions. As an example, SpiNNaker uses fixed-point number representation instead of floating point arithmetic as the default representation, which leads to programming difficulties and constraints such as the lack of native division operation and precision issues (Furber et al., 2013).

Integrating of PyNN into the design of NeuroFlow simulator offers a number of advantages in terms of model development and performance gain. PyNN shortens the time in model development, allowing model builders to port the simulation to a new simulation environment with less time and cross-check the correctness of their models in other simulators. It also allows automated customization of hardware which can optimize the utilization of hardware resources. Currently development with the PyNN framework faces a number of challenges, such as backward incompatibility, minor defects, and the lack of technical support; but the idea of integrating a high-level neural description language with a hardware platform for accelerating simulation is attractive and useful.

Neural networks for different purposes require various levels of abstractions and precisions. To date there is no consensus on what is the optimal level of abstraction to describe a biological neural network. Generally models with higher complexity have a richer dynamics which contributes to their higher computational capacity. For example, non-linear dendrites can solve linearly non-separable classification problems (Cazé et al., 2013), and extending point neurons to include one or more dendritic compartments can dramatically change the firing dynamics of the neuron, which can account for observations from experiments (Rospars and Lánský, 1993; Vaidya and Johnston, 2013). So changes to the model descriptions will affect the need for computational capability of a neurocomputer. While these properties are generally ignored when designing a neurocomputer, they are sometimes crucial in neural simulations. Given the large variety of neuronal, synaptic, and plasticity models used in neuroscience, inflexible neural simulation platforms would have limited value for computational neuroscientists.

In this regard, one possible use of NeuroFlow is for prototyping of neurocomputers. Engineers can test various configurations using FPGA-based neurocomputers and find the optimal precision for a given application and use the minimal complexity and precision as system configurations. Similar to ASIC prototyping using FPGAs, prototyping using NeuroFlow can reduce the cost of chip modification and can benefit future neural-based computation systems.

As illustrated in the experiments, NeuroFlow demonstrates good performance and flexibility, but it has a number of issues to address in order to broaden its appeal. While changes in neuronal, synaptic and simulation parameters only require generating new data files, FPGA requires considerable synthesis time to recompile the hardware configurations. However, there have been a number of attempts to reduce the synthesis time by using precompiled modules, such as hard macro stitching (Lavin et al., 2011) and modular reconfiguration (Sedcole et al., 2006). While the current system is efficient in handling a large network size of 10^5^–10^6^ neurons using simple interconnect, more work has to be done on the neural mapping scheme and intercommunication between nodes, such as similar work on SpiNNaker (Khan et al., 2008) to extend the scalability of the current system to support simulation of larger networks. Another issue is power consumption of the system. NeuroFlow make use of external DRAM and hence is not as power efficient as standalone FPGA platforms, but replacing them with faster and more power efficient SRAM can solve the problem.

Currently there is a major gap in our understanding between small neuronal networks and the neural system as a whole. Models such as the work by Izhikevich and Edelman (2008) incorporate the dynamics of individual neurons in a large-scale human brain model, which can be used to compare against whole-brain imaging data and can potentially lead to development of personalized diagnosis and treatments. Development of neural simulators such as NeuroFlow enables neuroscientists to study neural dynamics in high speed and explore the parameter space and fitting of parameters to neurophysiological measurements, which bridge the gap between the small and large-scale studies. NeuroFlow shows promising capability in neural simulation in performance, ease of use for neuroscientists and flexibility as demonstrated in the polychronous network and somatosensory cortex simulations. With the customizability offered by NeuroFlow, it facilitates exploration of various hardware configurations that may lead to better and more efficient neural implementations. There are also plans to extend functions of NeuroFlow, such as the inclusion of ball-and-stick models and AMPA, NMDA, and GABA synapses with non-linear temporal dynamics, in order to support simulation of more complex neural dynamics and make the platform useful for a broader group of neuroscientists.

## Author contributions

KC, SS, and WL conceived the idea for the study, KC carried out the study and prepared the figures, KC, SS, and WL wrote the manuscript. All authors reviewed the manuscript.

### Conflict of interest statement

The authors declare that the research was conducted in the absence of any commercial or financial relationships that could be construed as a potential conflict of interest.
